# Exploring the relationship between teaching autonomy and Chinese teachers' organizational commitment: a chain mediation model of self-efficacy and career well-being

**DOI:** 10.3389/fpsyg.2026.1820922

**Published:** 2026-04-29

**Authors:** Guangqiang Wang, Jinjin Bian, Huayang Zhang

**Affiliations:** 1 Department of Educational Management, Faculty of Education, East China Normal University, Shanghai, China; 2 School of Digital Media, Shenzhen Polytechnic University, Shenzhen, China

**Keywords:** career wellbeing, Chinese teacher, organizational commitment, self-efficacy, teaching autonomy

## Abstract

**Objective:**

This study aims to examine the relationship between teaching autonomy and teachers' organizational commitment based on the conservation of resources theory and to reveal the mediating roles of self-efficacy and career well-being in this relationship.

**Methods:**

The sample consisted of 2,996 teachers from the 2018 Teaching and Learning International Survey (TALIS) Shanghai database. Partial least squares structural equation modeling and the bias-corrected percentile Bootstrap method were employed for data analysis.

**Results:**

Teaching autonomy was significantly and positively correlated with teachers' organizational commitment, self-efficacy, and career well-being. Both self-efficacy and career well-being were significantly and positively related to teachers' organizational commitment. Self-efficacy was also significantly and positively associated with teachers' career well-being. Furthermore, self-efficacy and career well-being not only functioned as independent mediators between teaching autonomy and teachers' organizational commitment but also had a chain mediating role.

**Conclusion:**

This study reveals the mediating roles of self-efficacy and career well-being, thereby contributing to understanding the mechanisms through which teaching autonomy influences teachers' organizational commitment. Meanwhile, school administrators can enhance teachers' organizational commitment by increasing their teaching autonomy, self-efficacy, and career well-being.

## 1 Introduction

In the context of deepening educational reforms and the increasing prevalence of teacher turnover, teachers' organizational commitment is widely recognized as playing a crucial role in effective school management and in ensuring the stability of the education system (Amoah et al., 2021; Bogler and Berkovich, 2022). Teachers' organizational commitment refers to their psychological belonging and emotional attachment to the school, as well as their willingness to remain at the school based on these feelings (Chung, 2019). High levels of organizational commitment among teachers are advantageous for both individual and school development. Research showed that it was associated with higher performance (Van Waeyenberg et al., 2022), job satisfaction (Cayupe et al., 2023), work engagement (Dagdeviren Ertaş and Özdemir, 2024), organizational citizenship behaviors (Hermanto et al., 2024), and lower turnover intentions (Li et al., 2021). Given its importance, identifying the antecedents and underlying mechanisms that strengthen teachers' organizational commitment remains a central concern for educational researchers.

Currently, scholars have explored the effects of school environmental factors (e.g., organizational justice Donglong et al., 2020, transformational leadership Kilinç et al., 2024, and distributed leadership Bellibaş et al., 2024) and individual psychological factors (e.g., psychological empowerment Huang et al., 2021, job satisfaction Wang et al., 2020, and emotional intelligence Li et al., 2024) on teachers' organizational commitment. However, the relationship between teaching autonomy and teachers' organizational commitment has not yet received widespread attention. Teaching autonomy refers to the degree of control teachers have over their instructional matters (Xia et al., 2023). According to the conservation of resources theory (COR), individuals strive to acquire and retain various resources in the face of stress, including object, conditional, energetics and personal characteristic resources (Hobfoll, 1989). Within this framework, teaching autonomy is considered an important conditional resource that can help teachers alleviate work-related stress and foster a positive psychological state. Teaching autonomy is an important driving force in promoting teachers' professional development. When teachers have greater autonomy in the teaching process, they can adjust the content and methods of instruction according to their instructional needs. This sense of control significantly increases job satisfaction and effectively reduces work alienation and turnover intentions (Liu et al., 2021; Wang, 2025). Consequently, teaching autonomy may contribute to strengthening teachers' organizational commitment, warranting further exploration of the relationship between them.

Meanwhile, self-efficacy and career wellbeing, as important personal characteristic resources for teachers, may play mediating roles in the relationship between teaching autonomy and teachers' organizational commitment. From the perspective of the COR theory (Hobfoll, 2002), teaching autonomy, as a conditional resource, can facilitate teachers' access to personal characteristic resources (e.g., self-efficacy and career wellbeing), which in turn alleviates stress and enhances their organizational commitment. Specifically, teachers with high teaching autonomy are able to make flexible decisions about instructional design, pedagogy, and classroom management, which not only boosts their confidence in accomplishing their instructional tasks but also enhances their sense of meaning and wellbeing in their professional development (Pan et al., 2023; Peng et al., 2022). As teachers' self-efficacy and career wellbeing increase, their job satisfaction (Liu Y. et al., 2023; Ma and Marion, 2025) and work engagement also improve (Cai et al., 2022; Zeng et al., 2019), enabling them to maintain a strong sense of emotional belonging and loyalty to their schools and to demonstrate greater organizational commitment.

In summary, few studies have examined the relationship between teaching autonomy and teachers' organizational commitment. To address this gap, this study examines the relationship between teaching autonomy and teachers' organizational commitment based on the COR theory and reveals the mediating role of self-efficacy and career wellbeing between them. The study attempts to answer the following research questions:

RQ1: Does teaching autonomy correlate positively with teachers' organizational commitment?
RQ2: Do self-efficacy and career wellbeing mediate the relationship between teaching autonomy and teachers' organizational commitment?

## 2 Literature review and hypotheses

### 2.1 Teaching autonomy and teachers' organizational commitment

Organizational commitment refers to an individual's psychological attachment to a particular organization (Bogler and Berkovich, 2022) and encompasses three dimensions: affective, normative, and continuance (Meyer and Allen, 1991). Teacher organizational commitment reflects teachers' recognition, attachment, and loyalty to their school (Li et al., 2024). Teaching autonomy refers to a teacher's power to make independent decisions regarding instructional content, methods, and curriculum (Wang, 2025). As a positive job characteristic, teaching autonomy plays an important role in increasing teachers' organizational commitment. Previous research showed that job autonomy, as an important job resource, can effectively contribute to individual development (Bakker and Demerouti, 2017; Pan et al., 2024). Within the framework of the COR theory, teaching autonomy is conceptualized as a conditional resource that facilitates proactive resource investment while mitigating resource depletion, thereby enabling teachers to maintain a positive psychological state and demonstrate more positive behavioral performance. Specifically, higher levels of autonomy provide teachers with greater discretion and responsibility to make independent and flexible decisions about teaching styles and workflows (Hsieh et al., 2024), thereby fulfilling their psychological need for autonomy. This not only significantly increases job satisfaction (Liu et al., 2021) but also effectively alleviates negative emotions such as emotional exhaustion and promotes teachers' mental health (Peng et al., 2022; Wang et al., 2024b). Therefore, there may be a significant positive association between teaching autonomy and teachers' organizational commitment. Several studies provided empirical evidence for this inference. For example, in a study involving 1,278 university teachers, Huang et al. (2021) found that psychological empowerment was significantly and positively related to teachers' organizational commitment. Yao et al. (2024) showed that teacher empowerment significantly and positively affected teachers' affective commitment. Boudouaia et al. (2024) found a significant positive correlation between empowerment and English language teachers' commitment to their schools. Therefore, based on the above analysis, the following hypothesis is proposed:

H1: Teaching autonomy is significantly and positively related to teachers' organizational commitment.

### 2.2 The mediating effect of self-efficacy

Self-efficacy refers to an individual's belief in their ability to successfully achieve a particular goal (Bandura, 1977). In educational contexts, teacher self-efficacy is typically defined as a teacher's belief in their ability to successfully accomplish specific teaching tasks (Li, 2023). According to the COR theory, resources do not exist in isolation or stasis but are engaged in a dynamic process of constant flow and interaction (Hobfoll et al., 2018). This means that conditional resources (e.g., teaching autonomy) can be transformed into more intrinsic personal characteristic resources (e.g., self-efficacy), which can have a profound effect on an individual's psychological state (Hobfoll, 2002). When teachers possess a high level of autonomy in the teaching process, they can exercise greater control over their teaching activities and have more freedom to experiment with new instructional models and strategies to address various teaching challenges. This high degree of autonomy enables teachers to accumulate successful experiences in their teaching practice, significantly enhancing their self-efficacy (Peng et al., 2022). Research indicated that teacher autonomy was significantly and positively related to teachers' self-efficacy (Huang et al., 2024; Valckx et al., 2020). Therefore, a close link may exist between teaching autonomy and teachers' self-efficacy.

According to the COR theory, self-efficacy, as a personal characteristic resource, can help individuals effectively mobilize the necessary resources to alleviate stress responses (Halbesleben et al., 2014; Hobfoll, 1989). When teachers are confident in their ability to accomplish teaching tasks, they become more engaged in their work (Jiao et al., 2022) and proactively seek strategies to solve the problems they encounter. In this process, teachers experience a greater sense of job meaning and job satisfaction (Fang and Qi, 2023), and this positive work state leads to a significant increase in their commitment to their school, reducing the likelihood of turnover. Orgambídez et al. (2019) showed that self-efficacy not only directly predicted the organizational commitment of Spanish nurses but also indirectly influenced their organizational commitment through increased work engagement. Na-Nan et al. (2021) found a significant positive correlation between self-efficacy and organizational commitment among Thai employees. Cayupe et al. (2023) showed that self-efficacy was significantly and positively related to teachers' organizational commitment. Therefore, based on the above analysis, the following hypotheses are proposed:

H2a: Teaching autonomy is significantly and positively related to teachers' self-efficacy.
H2b: Self-efficacy is significantly and positively related to teachers' organizational commitment.
H2c: Self-efficacy mediates the relationship between teaching autonomy and teachers' organizational commitment.

### 2.3 The mediating effect of career wellbeing

Career wellbeing is defined as an individual's long-term satisfaction with career achievement, change, and sustainability in a complex work environment (Coetzee et al., 2021). Teachers' career wellbeing refers to their satisfaction and positive affective experiences related to their career development and teaching work, reflecting the degree of congruence between their career experiences and expectations (Liu J. et al., 2023; Wang et al., 2025a). In the COR theoretical perspective (Halbesleben et al., 2014; Hobfoll, 2002), teaching autonomy, viewed as a conditional resource, effectively supports teachers' access to personal characteristic resources, such as career wellbeing. Teaching autonomy empowers teachers to determine when and how to carry out their work (Wang, 2025), thus achieving a better fit between personal preferences and teaching tasks. This freedom and flexibility allow teachers to design and implement their activities more effectively, reducing unnecessary workloads. Consequently, this can alleviate job stress and burnout (Elrayah et al., 2023; Wang et al., 2024b), significantly enhancing career wellbeing. Furthermore, when teachers are granted autonomy, they feel the trust, respect, and support from school administrators, further improving their wellbeing. Research showed that autonomy is significantly and positively correlated with teachers' wellbeing (Lian-Beng et al., 2021). Therefore, teaching autonomy may also positively predict teachers' career wellbeing.

In the COR theoretical framework (Halbesleben et al., 2014; Hobfoll, 1989), career wellbeing is also viewed as a vital personal characteristic resource contributing to teachers' organizational commitment. As a positive affective experience (Coetzee et al., 2021), career wellbeing profoundly influences teachers' overall attitudes toward their jobs and the schools they work in. Teachers who report high levels of career wellbeing are more likely to invest energy and time into their teaching responsibilities and to view the school as a platform for realizing professional goals and personal values (Huang et al., 2023). This emotional investment strengthens their sense of belonging and identification with the school, increasing their willingness to contribute to its long-term development. Additionally, Hu et al. (2022) found a positive correlation between career wellbeing and teachers' career commitment in ethnic areas of China. Huang et al. (2023) demonstrated that career wellbeing was significantly and positively correlated with kindergarten teachers' career commitment. Li et al. (2024) indicated that psychological wellbeing significantly and positively predicted teachers' organizational commitment. Therefore, based on the above analysis, the following hypotheses are proposed:

H3a: Teaching autonomy is significantly and positively related to teachers' career wellbeing.
H3b: Career wellbeing is significantly and positively related to teachers' organizational commitment.
H3c: Career wellbeing mediates the relationship between teaching autonomy and teachers' organizational commitment.

### 2.4 The chain-mediating effect of self-efficacy and career wellbeing

There is a close relationship between self-efficacy and teachers' career wellbeing. Many scholars believe that self-efficacy is a critical predictor of individuals' wellbeing (Shao, 2023; Yin, 2022). Teachers with high self-efficacy are confident in their ability to plan and effectively accomplish their teaching tasks, actively setting milestones to address various challenges they encounter in the teaching process. This confidence allows them to be more proactive in facing difficulties and finding solutions, leading to a greater sense of job achievement and satisfaction (Fang and Qi, 2023). Additionally, teachers with high self-efficacy typically experience fewer negative emotions, such as stress and burnout (Chen et al., 2024; Hu et al., 2019), which helps them maintain positive work attitudes and mental health. Thus, from this perspective, teachers' self-efficacy may positively predict their career wellbeing. Numerous empirical studies support this view. Liang et al. (2022) found that teachers' teaching efficacy positively predicted their hedonic and eudaimonic wellbeing. Fu et al. (2022) showed that self-efficacy was significantly and positively related to the subjective wellbeing of Chinese special education teachers and mediated the relationship between social support and their wellbeing. In a study involving 375 Chinese secondary school English teachers, Wang et al. (2024a) found that self-efficacy not only directly and positively affected teachers' wellbeing but also indirectly through increased teaching satisfaction and resilience. Based on the above analysis, the following hypotheses are proposed:

H4a: Self-efficacy is significantly and positively related to teachers' career wellbeing.
H4b: Self-efficacy and career wellbeing have a chain mediating role between teaching autonomy and teachers' organizational commitment.

**Figure 1:**
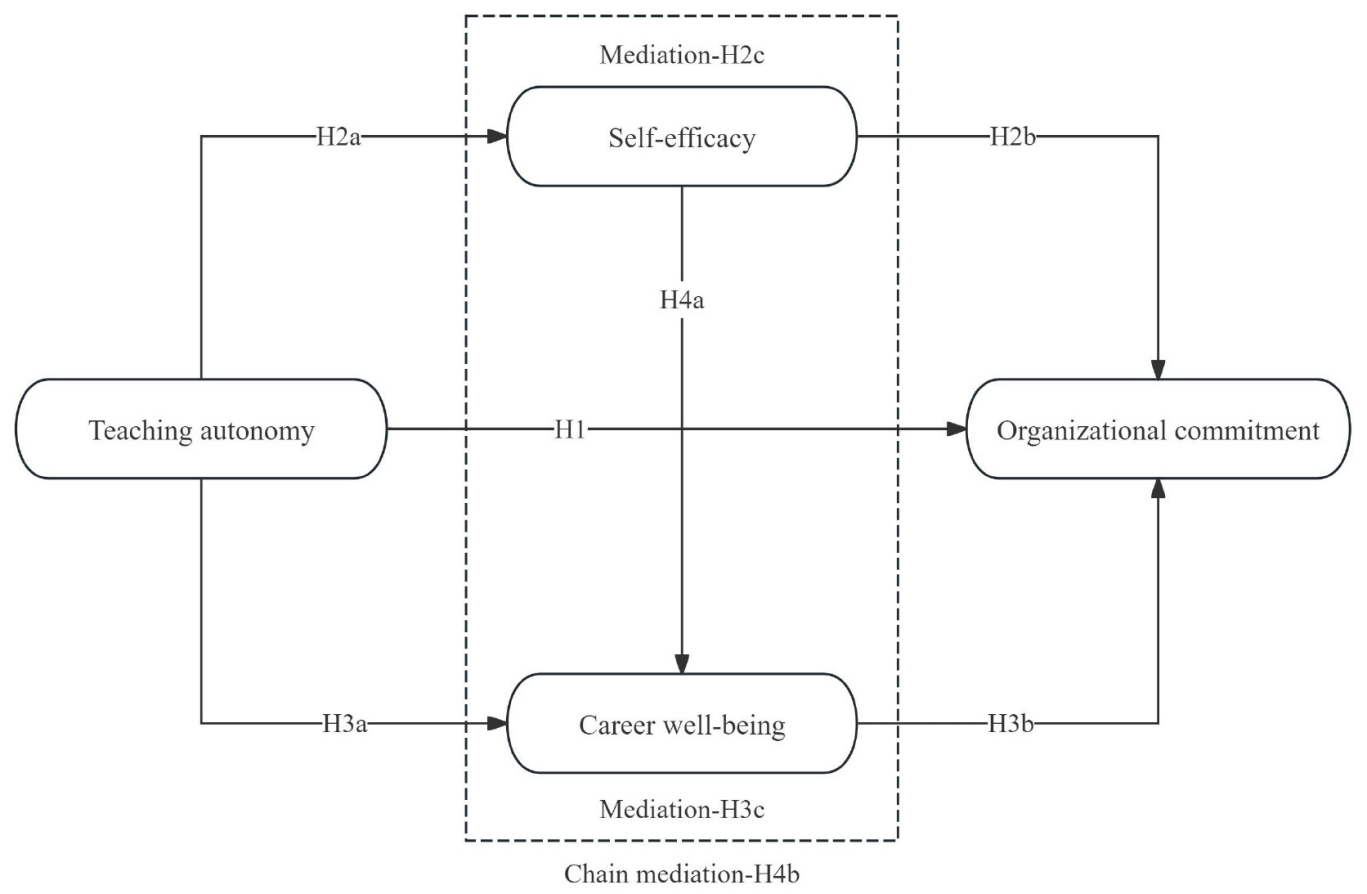
Research model.

Based on the study's purpose and the research hypotheses presented above, a research model is proposed, as illustrated in Figure 1.

## 3 Methods

### 3.1 Participant

The Organization for Economic Co-operation and Development (OECD) conducted the Teaching and Learning International Survey (TALIS), an international cross-sectional survey focusing on teacher development, in 2018. The survey encompasses numerous countries and regions, systematically collecting detailed data on teacher preparation, working conditions, professional development, and teaching practice. TALIS has garnered widespread global attention, and its database serves as a significant resource for education researchers and policymakers, facilitating insights into the current status and challenges of teachers' professional development and enabling the formulation of more effective education policies and improvement measures.

The TALIS 2018 Shanghai database was selected as the data source for the present study. A total of 3,976 junior high school teachers from Shanghai were surveyed in the database. Due to missing values in the database, certain samples were excluded during the data cleaning process to ensure the scientific rigor and accuracy of the data analysis, resulting in a final sample of 2,996 valid cases. Of the valid sample, 766 teachers (25.57%) were male, and 2,230 (74.43%) were female. Regarding educational background, 2,587 teachers (86.35%) held a bachelor's degree or less, while 409 (13.65%) held a master's degree or higher. The average teaching experience is 16.36 years.

### 3.2 Measures

#### 3.2.1 Teaching autonomy

Teaching autonomy was measured using the Teaching Autonomy Scale (TAS) from the teacher questionnaire. The TAS comprises five items, such as “Determining course content,” “Selecting teaching methods,” and “Assessing students' learning.” A four-point Likert scale was utilized, ranging from “1 = strongly disagree” to “4 = strongly agree,” with higher scores indicating greater levels of teacher autonomy. Wang and Bai (2025) showed that these items effectively assess teaching autonomy, exhibiting good reliability and validity.

#### 3.2.2 Organizational commitment

According to Bellibaş et al. (2024), organizational commitment was measured using three items from the teacher questionnaire, including “I would like to change to another school if that were possible,” “I enjoy working at this school,” and “I would recommend this school as a good place to work.” A four-point Likert scale was employed, ranging from “1 = strongly disagree” to “4 = strongly agree,” with higher scores indicating greater organizational commitment among teachers. Item TT3G53C was reverse-scored, while the remaining items were scored positively.

#### 3.2.3 Self-efficacy

Self-efficacy was measured using the Self-Efficacy Scale (SES) from the teacher questionnaire. The SES consists of three dimensions: self-efficacy in classroom management, self-efficacy in instruction, and self-efficacy in student engagement. It includes 12 items, such as “Use a variety of assessment strategies,” “Get students to follow classroom rules,” and “Make my expectations about student behavior clear.” A four-point Likert scale ranging from “1 = Not at all” to “4 = A lot” was utilized, with higher scores indicating greater teacher self-efficacy. It has been demonstrated by Teng et al. (2024) that these items are effective in assessing teacher self-efficacy, exhibiting good reliability and validity.

#### 3.2.4 Career wellbeing

According to Liu J. et al. (2023), career wellbeing was measured by eight items from the teacher questionnaire, such as “I think that the teaching profession is valued in society,” “Apart from my salary, I am satisfied with the terms of my teaching (e.g., benefits, work schedule),” and “Teachers are valued by the media in this country/region.” A four-point Likert scale was used, ranging from “1 = strongly disagree” to “4 = strongly agree,” with higher scores indicating higher levels of teachers' career wellbeing.

### 3.3 Data analysis

This study employs partial least squares structural equation modeling (PLS-SEM) to examine the relationships among teaching autonomy, career wellbeing, self-efficacy, and organizational commitment. A primary reason for this choice is that, similar to many studies, the dataset in this research deviates from a normal distribution, and PLS-SEM is better equipped to handle non-normally distributed data (Moisescu et al., 2025). Furthermore, this study examines not only the direct relationship between teaching autonomy and teachers' organizational commitment but also the mediating roles of self-efficacy and career wellbeing. PLS-SEM is particularly well-suited for managing such complex models (Moisescu et al., 2025). Liu J. et al. (2023) previously utilized PLS-SEM to examine the relationships among distributed leadership, self-efficacy, and wellbeing based on the TALIS Shanghai dataset. Consequently, PLS-SEM facilitates effective data analysis and hypothesis testing for this study.

First, SPSS 24.0 and AMOS 23.0 software are employed to conduct Harman's single-factor test and confirmatory factor analysis, respectively, to assess the presence of severe common method bias in the data, thereby ensuring the scientific validity and accuracy of subsequent analyses. Second, SPSS 24.0 is used to perform descriptive statistics and correlation analyses, thereby establishing a foundation for the mediation effect tests. Finally, SmartPLS 4.0 software is utilized to construct a PLS-SEM model to examine the relationships among teaching autonomy, self-efficacy, career wellbeing, and teachers' organizational commitment. Additionally, the bias-corrected percentile Bootstrap method (with 5,000 resamples) is employed to test the significance of the mediating roles of self-efficacy and career wellbeing. If the 95% confidence interval (CI) does not include zero, the mediating effects of self-efficacy and career wellbeing were deemed significant.

## 4 Results

### 4.1 Common method bias tests

This study assessed common method bias using the Harman single-factor test. The results indicated that the eigenvalues of the four extracted factors all exceeded 1.0, with the first factor accounting for only 33.38% of the variance, well below the critical value of 40%. Additionally, referencing prior research (Wang et al., 2025b), this study conducted a confirmatory factor analysis of the single-factor model. The results indicated that the model fit indices were χ^2^/df = 78.509, NFI = 0.512, RFI = 0.473, IFI = 0.515, TLI = 0.476, CFI = 0.515, RMSEA = 0.161, and SRMR = 0.166, which indicated a poor model fit. Therefore, it was concluded that no significant common method bias was present, providing a reliable basis for subsequent analyses.

### 4.2 Descriptive statistics and correlation analysis

Table 1 indicates that teaching autonomy was significantly and positively correlated with teachers' organizational commitment, self-efficacy, and career wellbeing (*r* = 0.199, *p* < 0.001; *r* = 0.379, *p* < 0.001; *r* = 0.145, *p* < 0.001). Self-efficacy was also significantly and positively correlated with both teachers' organizational commitment and career wellbeing (*r* = 0.243, *p* < 0.001; *r* = 0.215, *p* < 0.001). In addition, career wellbeing was significantly and positively correlated with teachers' organizational commitment (*r* = 0.607, *p* < 0.001).

**Table 1:** Descriptive statistics and correlation analysis.

Variable	M	SD	1	2	3	4
1 = Teaching autonomy	3.389	0.507	1			
2 = Organizational commitment	2.827	0.538	0.199^***^	1		
3 = Self-efficacy	3.298	0.536	0.379^***^	0.243^***^	1	
4 = Career wellbeing	2.682	0.517	0.145^***^	0.607^***^	0.215^***^	1

^***^p < 0.001 (two-tailed); M, mean; SD, standard deviation.

### 4.3 Measurement model assessment

According to Hair et al. (2021), the reliability, convergent validity, and discriminant validity of each construct in the structural model should be assessed. When both Cronbach's α coefficients and composite reliability (CR) exceed 0.70 (Moisescu et al., 2025), it indicates that these constructs demonstrate good internal consistency and composite reliability. When all factor loadings for each construct are greater than 0.60 (Sohail, 2023) and their average variance extracted (AVE) values exceed 0.50 (Moisescu et al., 2025), it signifies acceptable convergent validity. Table 2 demonstrates that the measurement model in this study exhibited strong internal consistency, composite reliability, and convergent validity.

**Table 2:** Reliability and validity assessment.

Constructs	Items	Factor loading	Cronbach's alpha	CR	AVE
Teaching autonomy	TA1	0.812	0.919	0.939	0.756
	TA2	0.907			
	TA3	0.895			
	TA4	0.864			
	TA5	0.866			
Organizational commitment	OC1	0.672	0.727	0.845	0.649
	OC2	0.866			
	OC3	0.863			
Self-efficacy	SE1	0.787	0.951	0.957	0.648
	SE2	0.782			
	SE3	0.81			
	SE4	0.831			
	SE5	0.825			
	SE6	0.806			
	SE7	0.822			
	SE8	0.801			
	SE9	0.805			
	SE10	0.805			
	SE11	0.805			
	SE12	0.781			
Career wellbeing	CWB1	0.748	0.858	0.889	0.502
	CWB2	0.638			
	CWB3	0.737			
	CWB4	0.622			
	CWB5	0.664			
	CWB6	0.778			
	CWB7	0.689			
	CWB8	0.775			

CR, composite reliability; AVE, average variance extracted.

Meanwhile, discriminant validity has been further assessed. First, the Fornell-Larker criterion was applied. Table 3 demonstrates that the square root of the AVE for each variable was greater than the correlation coefficient between that variable and the others (Moisescu et al., 2025). Second, according to Henseler et al. (2015), the heterotrait-monotrait (HTMT) ratio is employed to assess discriminant validity. Table 4 reveals that all HTMT values were below the 0.85 threshold (Moisescu et al., 2025). Therefore, the measurement model exhibits good discriminant validity.

**Table 3:** Fornell-Larcker criterion.

Constructs	1	2	3	4
1 = Teaching autonomy	0.870			
2 = Organizational commitment	0.196	0.805		
3 = Self-efficacy	0.384	0.244	0.805	
4 = Career wellbeing	0.157	0.637	0.223	0.709

**Table 4:** HTMT ratio.

Constructs	1	2	3	4
1 = Teaching autonomy				
2 = Organizational commitment	0.243			
3 = Self-efficacy	0.409	0.291		
4 = Career wellbeing	0.168	0.779	0.24	

### 4.4 Hypothesis testing

This study assesses the explanatory power and predictive ability of the structural model. First, the model's standardized root mean square residual (SRMR) value is 0.058, demonstrating that it is below the 0.08 threshold. Second, the *R*^2^ values for self-efficacy, career wellbeing, and organizational commitment are 0.147, 0.056, and 0.420, respectively. Meanwhile, their *Q*^2^ values are recorded as 0.146, 0.024, and 0.037, respectively. Therefore, the model fits well, falls within an acceptable range, and exhibits a certain degree of explanatory and predictive power.

**Figure 2:**
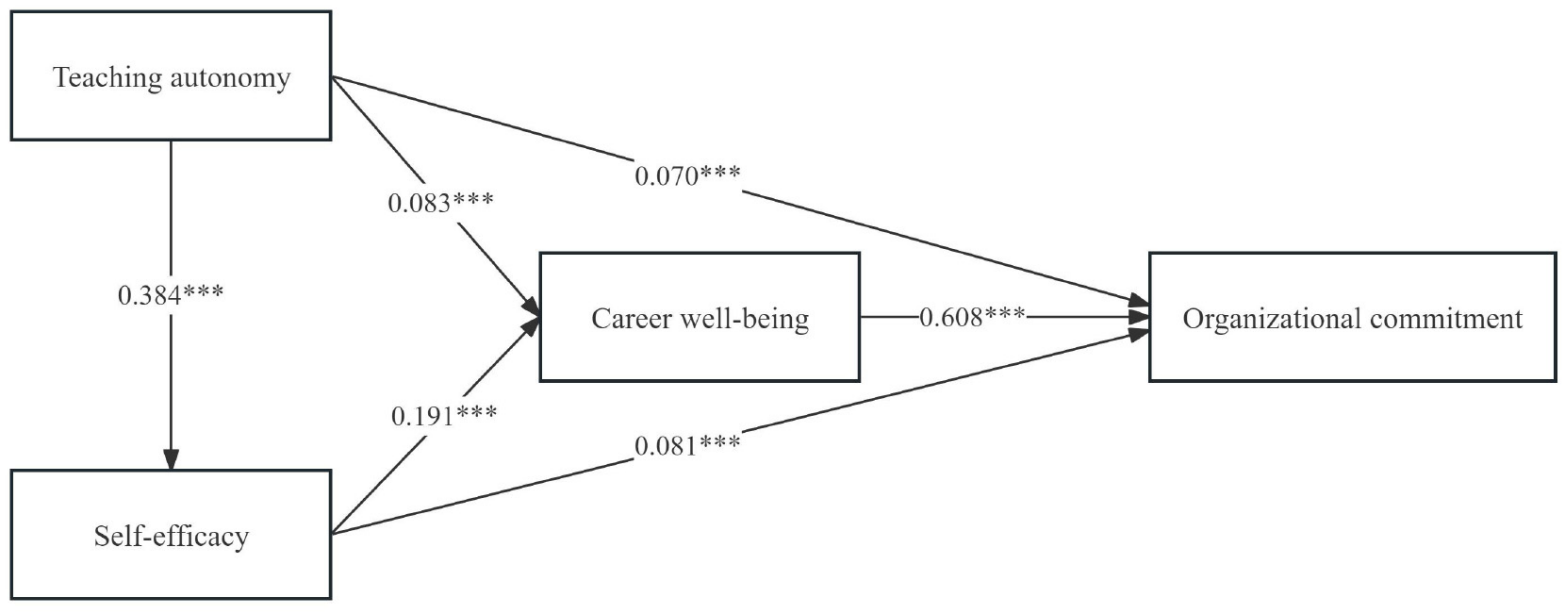
Structural model. ^***^p < 0.001.

This study develops a PLS-SEM model and employs the bias-corrected percentile bootstrap method (5,000 resamples) to test the hypotheses. Figure 2 indicates that teaching autonomy was significantly and positively associated with teachers' organizational commitment (β = 0.070, *p* < 0.001), self-efficacy (β = 0.384, *p* < 0.001), and career wellbeing (β = 0.083, *p* < 0.001). Self-efficacy was significantly and positively correlated with teachers' organizational commitment (β = 0.081, *p* < 0.001) and career wellbeing (β = 0.191, *p* < 0.001). Career wellbeing was significantly and positively related to teachers' organizational commitment (β = 0.608, *p* < 0.001). Therefore, hypotheses H1, H2a, H2b, H3a, H3b, and H4a were supported.

Table 5 indicates that the direct effect of teaching autonomy on teachers' organizational commitment was 0.070, with a 95% CI that did not include zero, indicating a significant direct effect. The mediating effect of self-efficacy was 0.031, with a 95% CI that excluded zero, suggesting that self-efficacy significantly mediates the relationship between teaching autonomy and teachers' organizational commitment. The mediating effect of career wellbeing was 0.050, with a 95% CI that did not include zero, indicating that career wellbeing also mediates the relationship between teaching autonomy and teachers' organizational commitment. Furthermore, the chain mediation effect through self-efficacy and career wellbeing was 0.045, with a 95% CI excluding zero, suggesting that self-efficacy and career wellbeing have a chain mediation effect between teaching autonomy and teachers' organizational commitment. Therefore, hypotheses H2c, H3c, and H4b were confirmed.

**Table 5:** Total, direct, and mediation effects of self-efficacy and career wellbeing.

Path	Effect	SE	Bias-corrected 95% CI		Ratio
			Lower	Upper	
Total effect	0.196	0.019	0.158	0.231	
Direct effect	0.070	0.016	0.040	0.101	35.71%
Total indirect effect	0.126	0.013	0.100	0.152	64.29%
TA → SE → OC	0.031	0.006	0.019	0.043	15.82%
TA → CWB → OC	0.050	0.012	0.027	0.074	25.51%
TA → SE → CWB → OC	0.045	0.005	0.034	0.056	22.96%

TA, teaching autonomy; OC, organizational commitment; SE, self-efficacy; CWB, career wellbeing.

## 5 Discussion

Among the many factors influencing teachers' organizational commitment, the role of teaching autonomy has often been overlooked, and its mechanism of influence remains insufficiently explored. Consequently, grounded in the COR theory, this study examined the relationship between teaching autonomy and teachers' organizational commitment and revealed the mediating roles of self-efficacy and career wellbeing. By systematically testing the research hypotheses, several significant results were obtained, which are discussed in detail below.

First, the results indicated teaching autonomy was significantly and positively correlated with teachers' organizational commitment. This suggests that higher levels of teaching autonomy correspond to higher levels of organizational commitment among teachers. This finding aligns with the explanation provided by COR theory. From the perspective of the COR theory (Halbesleben et al., 2014; Hobfoll, 1989), teaching autonomy, as a conditional resource, can effectively reduce resource consumption and alleviate job stress while enhancing teachers' emotional identification and sense of belonging to the school, thereby increasing organizational commitment. Furthermore, this result is consistent with findings from similar previous studies. For example, Jain and Duggal (2018) found that job autonomy not only positively affected Indian employees' organizational commitment but also mediated the relationship between transformational leadership and their organizational commitment. Cheng and Jiang (2022) also reported that job autonomy was significantly and positively associated with Chinese social workers' organizational commitment. In the Chinese cultural context, teachers are especially sensitive to perceived organizational support and to changes in their power status due to collectivist values and high power distance. By granting greater teaching autonomy, schools not only provided teachers with more opportunities to plan and implement instructional activities (Xia et al., 2023), but more importantly, demonstrated institutional trust and recognition of teachers' professional competence (Peng et al., 2022). This recognition further stimulated teachers' sense of responsibility and mission, motivating their willingness to invest in the school's long-term development and maintain higher organizational commitment. In conclusion, the findings offer empirical support for the positive relationship between teaching autonomy and teachers' organizational commitment, thereby contributing to the advancement of related research.

Second, the results showed that self-efficacy mediated the relationship between teaching autonomy and teachers' organizational commitment, establishing the path: “teaching autonomy → self-efficacy → teachers' organizational commitment.” This finding implies that teaching autonomy not only directly and positively predicts teachers' organizational commitment but also indirectly impacts it by enhancing self-efficacy. On the one hand, teaching autonomy was significantly and positively associated with teachers' self-efficacy. This finding aligns with the explanation provided by the COR theory. According to the resource flow perspective of the COR theory (Halbesleben et al., 2014; Hobfoll, 2002), conditional resources (e.g., teaching autonomy) are initially transformed into personal characteristic resources (e.g., self-efficacy), which subsequently affect an individual's stress response and behavioral performance. Moreover, this result is consistent with similar previous studies (Huang et al., 2024; Peng et al., 2022). Particularly in school management systems that emphasize authority and hierarchy, administrative structures often limit teachers' autonomy, compelling them to adhere strictly to curriculum standards and administrative directives (Lundström, 2015). In such an environment, acquiring a degree of teaching autonomy is crucial, as it enables teachers to receive more positive feedback and experience a greater sense of accomplishment in the teaching process. This positive teaching experience not only enhances teachers' self-efficacy but also strengthens their commitment to the school, thereby fostering a virtuous cycle. On the other hand, the results indicated a significant and positive relationship between self-efficacy and teachers' organizational commitment. This finding is consistent with similar previous research (Chung, 2019; Dagdeviren Ertaş and Özdemir, 2024). From the COR theory perspective, self-efficacy, as a critical personal resource (Halbesleben et al., 2014; Hobfoll, 2002), plays a pivotal role in enhancing teachers' organizational commitment. In the process of adapting to complex school environments, numerous challenges and pressures are encountered by teachers. Teachers with high self-efficacy are more likely to proactively adopt multiple strategies to address challenges rather than retreat or withdraw. Positive feedback from successful problem-solving encourages teachers to continue developing within the school and strengthens their commitment to the institution. Thus, these findings underscore the significant role of self-efficacy in the process by which teaching autonomy influences teachers' organizational commitment and provide insights for related research.

Third, the results showed that career wellbeing mediated the relationship between teaching autonomy and teachers' organizational commitment. Specifically, the pathway “teaching autonomy → career wellbeing → teachers' organizational commitment” was established. This finding suggests that teaching autonomy not only directly and positively predicts teachers' organizational commitment but also indirectly influences it by enhancing career wellbeing. On the one hand, teaching autonomy was found to be significantly and positively associated with teachers' career wellbeing. This result is consistent with the explanation provided by the COR theory. According to the COR theory (Halbesleben et al., 2014; Hobfoll, 2002), teaching autonomy effectively enriches teachers' resources, enabling the investment in and acquisition of new resources, such as career wellbeing, while preserving existing resources. Furthermore, this finding aligns with results from similar previous studies (Wan et al., 2024; Wu et al., 2015). In educational contexts, teaching autonomy, as an important job characteristic, not only directly affects the quality and effectiveness of instruction (Hsieh et al., 2024) but is also closely related to teachers' job satisfaction (Liu et al., 2021). When teachers have greater teaching autonomy, they can better control the teaching process (Wang, 2025). This sense of mastery reduces the negative impact of external pressures and increases psychological resilience, enabling greater adaptability when facing challenges, which in turn facilitates the maintenance of positive emotions and greater wellbeing. On the other hand, it was found that career wellbeing was significantly and positively correlated with teachers' organizational commitment. This finding is consistent with similar previous research (Huang et al., 2023; Li et al., 2024). As a psychological resource, career wellbeing enhances teachers' identification with and reliance on the school. Teachers with high wellbeing are more likely to align their personal educational philosophies with the organizational goals of the school, thereby demonstrating a stronger sense of responsibility and moral obligation. This intrinsic identification significantly enhances their loyalty and commitment to the school, increasing their willingness to invest greater time and energy in advancing the institution. In conclusion, these findings highlight the crucial role of career wellbeing in the process by which teaching autonomy influences teachers' organizational commitment and offer guidance for effectively enhancing organizational commitment.

Finally, the results indicated that self-efficacy and career wellbeing played a chain-mediating role between teaching autonomy and teachers' organizational commitment, i.e., the pathway “teaching autonomy → self-efficacy → career wellbeing → teachers' organizational commitment” was established. As shown in Table 2, the chain-mediating effect of self-efficacy and career wellbeing accounted for 21.36% of the total effect, reflecting the importance of this path in the process through which teaching autonomy influences teachers' organizational commitment. Self-efficacy was found to be significantly and positively correlated with teachers' career wellbeing. This result is consistent with similar studies (Liang et al., 2022; Pan et al., 2022), which demonstrated that higher levels of teachers' self-efficacy are associated with increased wellbeing. When teachers have high self-efficacy, they make positive self-evaluations of their abilities and develop strong beliefs (Gordon et al., 2023). This self-confidence enables them to cope with various setbacks and challenges in their work and career development with greater ease, thereby effectively avoiding negative emotions such as burnout and anxiety (Fallah et al., 2023; Li, 2023) and enhancing their career wellbeing. Particularly in collectivist cultures, teachers' self-efficacy is reflected in their sense of identity and responsibility within the collective. Teachers with high self-efficacy are more likely to take collective responsibility and actively engage in teamwork. When they feel valued and supported within the collective, this sense of belonging significantly enhances their career wellbeing.

## 6 Theoretical and practical implications

This study makes a significant theoretical contribution to the existing literature. First, although teaching autonomy is recognized as a key factor influencing teachers' professional development, its specific relationship with organizational commitment has not been empirically examined. From a resource perspective, this study argued that teaching autonomy, as an important conditional resource, significantly enhanced teachers' organizational commitment. Through empirical analysis, we verified the positive relationship between teaching autonomy and teachers' organizational commitment, providing valuable insights for related research. Second, the mechanism by which teaching autonomy promotes teachers' organizational commitment has not been thoroughly explored. To address this gap, this study identifies self-efficacy and career wellbeing as key mediating variables based on the COR theory, thereby deepening the understanding of how teaching autonomy effectively promotes teachers' organizational commitment. In conclusion, this study offers a robust analytical framework for understanding the relationship between teaching autonomy and teachers' organizational commitment, providing a new perspective for exploring teachers' organizational commitment.

This study has important practical implications. First, school administrators should prioritize granting teachers' greater autonomy in their instructional practices. This process includes establishing empowerment mechanisms that enable teachers' to adjust instructional content and pacing based on students' actual learning needs. By forming teacher-led instructional design teams, teachers' can have greater space and freedom in planning their teaching content and selecting methods. Additionally, reducing unnecessary administrative interventions and excessive assessment pressure would allow teachers' to take greater control of their instructional activities. Second, various strategies should be adopted by school administrators to enhance teachers' self-efficacy. For instance, teacher-apprenticeship programs and collaborative teaching teams can be implemented to foster professional dialogue and collaboration, enhancing their confidence in teaching. Furthermore, creating a blended teacher training system that combines online and offline opportunities will help continuously improve teachers' professional competence and bolster their self-efficacy. Third, teachers' career wellbeing is crucial for effectively enhancing their organizational commitment. Administrators should streamline teachers' workloads to reduce pressure and prevent excessive fatigue. Regular workshops focused on teachers' mental health are also recommended to aid in emotional regulation and psychological resilience. It is advisable to encourage teachers' to participate in interest clubs and team activities to promote emotional connections and a sense of belonging among teachers'.

## 7 Limitations and future research

Several limitations should be acknowledged in this study. First, as cross-sectional data were used to examine the relationships among teaching autonomy, teachers' organizational commitment, self-efficacy, and career wellbeing, causal inferences cannot be drawn. Therefore, it is recommended that future research employ experimental or longitudinal designs to analyze the dynamic relationships among these variables in greater depth and to provide more robust evidence. Second, grounded in the COR theory, this study revealed the mediating roles of self-efficacy and career wellbeing in the relationship between teaching autonomy and teachers' organizational commitment. However, additional mediating variables may exist in the process by which teaching autonomy influences teachers' organizational commitment. Therefore, it is suggested that future studies further investigate these potential mediators to achieve a more comprehensive understanding of the mechanisms underlying the effects of teaching autonomy. Finally, this study examined the relationships among these variables in the Chinese context using TALIS Shanghai teacher data, which significantly limits the generalizability of the findings. It is recommended that future research validate these findings in Western cultural contexts and conduct international comparative analyses.

## Data Availability

The datasets presented in this study can be found in online repositories. The names of the repository/repositories and accession number(s) can be found at: https://www.oecd.org/en/data/datasets/talis-2018-database.html.
